# Bithiophene as a Sulfur-Based Promotor for the Synthesis of Carbon Nanotubes and Carbon-Carbon Composites

**DOI:** 10.3390/ijms24076686

**Published:** 2023-04-03

**Authors:** Alisa R. Bogdanova, Dmitry V. Krasnikov, Eldar M. Khabushev, Javier A. Ramirez B., Albert G. Nasibulin

**Affiliations:** Center for Photonic Science and Engineering, Skolkovo Institute of Science and Technology, Nobel Str. 3, 121205 Moscow, Russiad.krasnikov@skol.tech (D.V.K.);

**Keywords:** carbon nanotubes, catalyst promotor, sulfur, aerosol CVD, bitihiophene

## Abstract

We assess bithiophene (C_8_H_6_S_2_) as a novel sulfur-based promotor for the growth of single-walled carbon nanotubes (SWCNTs) in the aerosol (floating catalyst) CVD method. Technologically suitable equilibrium vapor pressure and an excess of hydrocarbon residuals formed under its decomposition make bithiophene an attractive promoter for the production of carbon nanotubes in general and specifically for ferrocene-based SWCNT growth. Indeed, we detect a moderate enhancement in the carbon nanotube yield and a decrease in the equivalent sheet resistance of the films at a low bithiophene content, indicating the improvement of the product properties. Moreover, the relatively high concentrations and low temperature stability of bithiophene result in non-catalytical decomposition, leading to the formation of pyrolytic carbon deposits; the deposits appear as few-layer graphene structures. Thus, bithiophene pyrolysis opens a route for the cheap production of hierarchical composite thin films comprising carbon nanotubes and few-layer graphene, which might be of practical use for hierarchical adsorbents, protective membranes, or electrocatalysis.

## 1. Introduction

Carbon nanotubes are a class of materials with a wide range of accessible properties [1]. The group of promising and cutting-edge applications of carbon nanotubes heavily depends on the targeted optimization of a specific set of properties optimized for each case [2,3,4]. Nevertheless, the industrial production of carbon nanotubes with tailored characteristics and “affordable” prices is still a challenge due to the high cost of postprocessing to achieve a specific chirality [5,6] and the lack of a general model for controlled synthesis [7,8]. The challenge results in extensive experimental studies using both classical [9,10] and machine-learning methods [11,12] to provide a multiparameter optimization for synthesis productivity (yield), along with purity, quality, conductivity, uniformity, etc. [13,14,15].

Besides the classical optimization of the reactor geometry, carbon source, catalyst nature, and temperature profile, relatively small portions of specific species (promotors) enhance carbon nanotube growth [16]. Sulfur-based volatile species are one of the most abundant promotors for carbon nanotube synthesis [17]. The sulfur addition accelerates carbon source decomposition [18] and lowers the melting point of iron-based catalysts [19], as sulfur usually accumulates in the catalyst’s vicinity [20]. Though the exact role of sulfur is rather complex and yet to be revealed, it is generally accepted that the introduction of an optimal amount of an S-containing compound enhances the yield and increases the nanotube diameter [17,18,21]. Thiophene, carbon disulfide, and sulfur have already been actively used as volatile sulfur compounds for carbon nanotube synthesis [16]. Nevertheless, a deep understanding of sulfur in carbon nanotube growth still poses a challenge, providing room for further improvement for emerging applications in energy transition [22,23], waste treatment [24,25], and selective adsorption [26].

**Figure 1:**
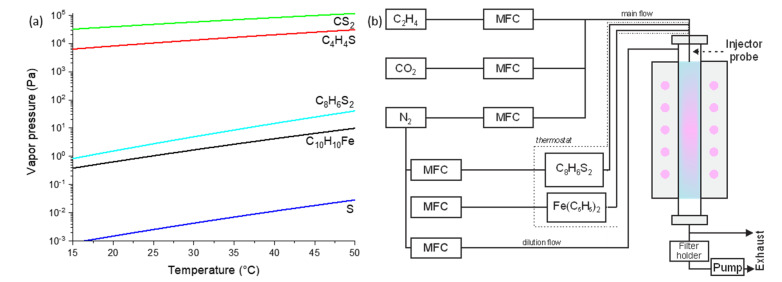
(**a**) Equilibrium vapor pressures of 2,2′-bithiophene (C_8_H_6_S_2_; cyan line), thiophene (C_4_H_4_S; red), sulfur (S; blue), carbon disulfide (CS_2_; green), and ferrocene (FeC_10_H_10_; black), [36]; (**b**) a scheme for the aerosol CVD reactor used in the work (MFC stands for a mass-flow controller; chemical formulas for the vessels/units filled with corresponding species).

Here, we propose and examine a novel sulfur-based compound—2,2′-bithiophene—as it provides a few advantages. An extended amount of hydrocarbon residuals (compared to sulfur and carbon disulfide) might positively contribute to catalyst activation and carbon nanotube growth. Indeed, we recently compared Fe-based catalysts with (ferrocene) and without (spark-discharge generated particles) hydrocarbon residuals [27]. The catalyst derived from ferrocene showed an increased productivity and different pattern for interaction with an etchant (CO_2_), most likely due to hydrocarbon residuals. Moreover, 2,2′-bithiophene is a volatile solid compound with a convenient equilibrium vapor pressure (Figure 1a) comparable to that of ferrocene. Thus, bithiophene can be easily incorporated into the aerosol CVD reactor based on CO, methane, or ethylene (unlike the need for an extremely precise dosage of pure thiophene, carbon disulfide, or high-temperature lines for sulfur) [8]. To the best of our knowledge, bithiophene has never been utilized as a promotor for carbon nanotube growth.

## 2. Results

We employ the aerosol (version of a floating catalyst method with an extreme bed dilution) CVD synthesis [28] (Figure 1b) of single-walled carbon nanotubes (SWCNTs) based on ethylene catalytic decomposition at 1000 °C. Briefly, ferrocene served as a source for Fe-based catalyst particles, ethylene as a carbon source, carbon dioxide as an etching agent [29], nitrogen as a carrier gas, and bithiophene as a promotor. Volatile ferrocene and bithiophene (Figure 1a) were transferred to the hot zone of the reactor with the nitrogen flow via the cartridges in a thermostat.

To provide a comprehensive assessment, we employed the following set of parameters. The reactor yield reflects the process productivity and corresponds to the area of SWCNT films with 90% transmittance at 550 nm per liter of gas [13]. The quality of carbon nanotubes is a vague parameter comprising a vast set of parameters. We use the generally accepted ratio of intensities for G and D modes of Raman spectra as a figure of quality (as the G band corresponds to vibrations of graphitic lattice, while the D band appears only for distortions in the ideal lattice) [30,31,32,33]. The equivalent sheet resistance serves as a key performance index for transparent conductors [34], indirectly describes the length, defectiveness, and diameter of carbon nanotubes [35], and corresponds to the sheet resistance of a film with 90% transmittance at 550 nm.

**Figure 2:**
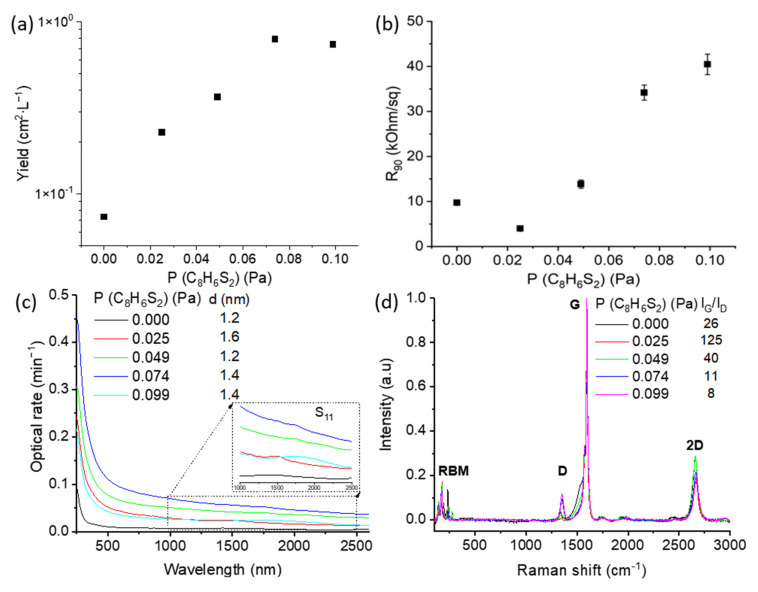
(**a**) Influence of bithiophene on SWCNT deposition rate, (**b**) equivalent sheet resistance, (**c**) UV-vis-NIR, and (**d**) Raman spectra for SWCNTs obtained with different bithiophene contents; RBM stands for radial breathing modes—a fingerprint of SWCNTs.

Similar to conventional sulfur-based promotors (acting via changes in the catalyst and carbon-source decomposition rate), the addition of 2,2′-bithiophene (C_8_H_6_S_2_) dramatically increases the yield of the process (Figure 2a). We believe the role of bithiophene to be close to that of thiophene, as it is one of the main products of decomposition [37]: changes in the catalyst melting point and carbon-source decomposition yield [18,19]. Moreover, we observe a slight increase in the nanotube diameter at low concentrations, similar to the literature [21]. It should be noted that the main feature of the aerosol CVD method—extreme dilution of the catalyst—limits a direct observation of the process as well as an assessment of the ppm level of sulfur residuals that might be in the reactor downstream. 

It is worth mentioning that at the lowest bithiophene concentrations, the equivalent sheet resistance demonstrated a two-fold improvement and subsequent increase with the promoter concentration (Figure 2b). Such a trend for the yield (process productivity) and equivalent sheet resistance (resistance at a certain film thickness) might be attributed to an increase in the nanotube length under low concentrations, followed by the enhancement of the catalyst activation [35]. This hypothesis agrees well to some extent with neglectable changes in the observed SWCNT diameter (Figure 2c) and a decrease in defectiveness under the lowest values only (Figure 2d). Nevertheless, the TEM studies proved that the hypothesis was wrong.

**Figure 3:**
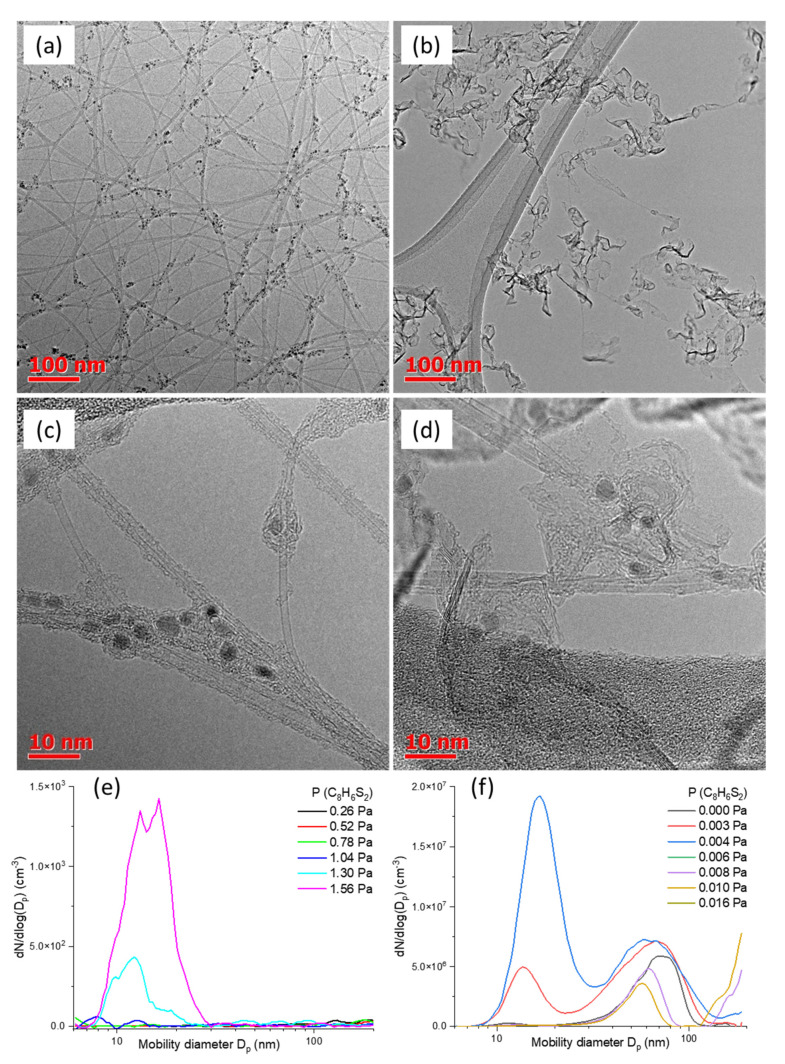
Typical TEM images of carbon nanotubes produced (**a**,**c**) without bithiophene and (**b**,**d**) with 0.08 Pa of bithiophene. DMA number size distributions of aerosol particles produced when bithiophene is supplied (**e**) in pure nitrogen and (**f**) in the ferrocene-CO mixture adjusted for SWCNT growth with the same residence time at 1000 °C.

TEM images (Figure 3) indicate that the presence of bithiophene results in the formation of an additional carbon nanomaterial: carbon flakes, i.e., few-layer graphene. The flake size varies within a wide range: from several nm to sub-micron values. The TEM observation of the few-layer graphene flakes also coincides with a decrease in the ratio of intensities of the 2D and G modes of Raman spectra (Figure 2d). Most likely, at a certain concentration, bithiophene decomposes non-catalytically (pyrolysis process) to produce carbon deposits that enhance the apparent yield. Indeed, the surface pyrolysis usually starts earlier than the volume one due to decreased activation energy [38].

It should be noted that most of the observed iron nanoparticles correspond to the non-activated catalyst particles, while the direct observation of a catalyst on the tip of SWCNT is limited to size and catalyst abundance [39]. We also predominantly observe single-walled carbon nanotubes (which coincides with the appearance of radial breathing modes in the Raman spectra; Figure 2d). At the same time, the direct diameter assessment is limited due to carbon nanotube bundling.

Interestingly, as it is one of the byproducts of thiophene pyrolysis, 2,2′-bithiophene appears to be less stable than usual sulfur promotors [37]. Indeed, 2,2′-bithiophene is one of the dominant byproducts of thiophene pyrolysis under temperatures of ca. 830 °C [37]. Nevertheless, it promptly disappears from the group of products at T > 900 °C. Moreover, the authors claim that the bithiophene concentration is heavily susceptible to the concentration of radical chain initiators [37]. As ferrocene is known to decompose at 400–600 °C [40,41,42] and ethylene at T > 800 °C, the reaction mixture might serve as such a radical chain initiator, i.e., hydrocarbon residuals that might promote bithiophene pyrolysis. Indeed, studies of particle size distributions with DMA clearly show that bithiophene pyrolysis occurs (Figure 3e) at partial pressures (>1 Pa; we did not observe any pyrolysis at lower temperatures, while the process is drastically enhanced at 1100 °C, even at 0.3 Pa). This is substantially higher than in the case of the SWCNT synthesis. Interestingly, even the smallest additions (Figure 3f) of bithiophene result in the formation of small aerosol particles (~15 nm), while the subsequent increase diminishes both the concentration and effective size of nanotubes (40–100 nm range) with a certain increase of the indistinguishable tail of large particles out of the device’s range. Though the use of hydrogen as a carrier gas might be a possible action to prevent bithiophene pyrolysis, the low-temperature stability of bithiophene limits its performance as a promotor of SWCNT growth.

We wish to stress that the obtained SWCNT/few-layer graphene composites might be of significant interest for various applications [43]. For example, such hybrid membranes should provide increased performance as EUV pellicles [44] or X-ray protective membranes. The high conductivity of materials combined with their morphology, which usually corresponds to an increased amount of surface, might also be attractive for electrocatalytic applications [45,46].

## 3. Materials and Methods

We employ the aerosol CVD synthesis (Figure 1b) of single-walled carbon nanotubes (SWCNTs) based on ethylene catalytic decomposition at 1000 °C. Ferrocene (98%, Sigma Aldrich, St. Louis, MO, USA) was used as a catalyst precursor (0.17 Pa), nitrogen (99.999%) as a carrier gas, ethylene (99.9%) as a carbon source (0.22 vol%), and small amounts of CO_2_ (99.995%) were utilized (0.20 vol%) to enhance the SWCNT synthesis [35]. According to SEM studies, the synthesis results in SWCNTs with a mean geometric length of 7 μm. Similar to ferrocene, 2,2′-bithiophene (C_8_H_6_S_2_, 98%, Sigma Aldrich) was carried out with a nitrogen gas flow out of a thermostat cartridge. The SWCNT aerosol was then collected on a filter (HAWP, Merck Millipore, Burlington, MA, USA) for the subsequent characterization. We assessed the SWCNT diameter distribution and yield utilizing UV-vis-NIR spectroscopy (Perkin Elmer Lambda 1050, Waltham, MA, USA); the latter was described elsewhere [13]. In brief, the yield corresponds to the area of the films with 90% transparency (at 550 nm) collected by passing a liter of the gas mixture. We examined the quality of the product by measuring the ratio of G to D mode intensities with Raman spectroscopy (Horiba LabRAM HR Evolution system, Kyoto, Japan) and by observing the morphology and structure of the produced material by transmission electron microscopy (FEI Tecnai G2 F20, Hillsboro, OR, USA). Furthermore, using four-probe measurements (Jandel RM3000, Leighton Buzzard, UK), we evaluated the equivalent sheet resistance: sheet resistance for a film with 90% transmittance in the middle of the visible region—a key performance indicator for transparent electrodes. Lastly, we observed the effective number size distributions of aerosols with a differential mobility analyzer (DMA; Scanning Mobility Particle Sizer Spectrometer 3938, Shoreview, MN, USA). It should be stressed that, strictly speaking, aerosol spectrometry provides the distribution of aerosol particles based on their electrical mobilities. Then, the mobility is transformed into a size, assuming a spherical shape for aerosol particles and taking into account the equilibrium charge state of the particle. Though this assumption is valid to some extent for non-activated catalyst particles (spherical shape or grape-like agglomerates), carbon nanotubes endure a complex quasi-cylindrical shape (in general, a nanotube or nanotube agglomerate is at least curved or has an even more complex geometry). This is why we refer to the parameter as an effective or mobility diameter. The usual values for the effective diameter of nanotube aerosols lie in the range of 40–100 nm.

## 4. Conclusions

In conclusion, for the first time, we assessed 2,2′-bithiophene (C_8_H_6_S_2_) as a novel sulfur-based promotor for SWCNT growth in an aerosol CVD reactor. We detected an enhancement in the carbon nanotube yield and a decrease in the equivalent sheet resistance of films under a low partial pressure of 0.025 Pa (the molar ratio of Fe to S was 3.4), indicating the improvement of the product properties. Relatively high concentrations and low temperature stability of bithiophene resulted in a non-catalytical decomposition (most likely owing to the ferrocene/ethylene mixture acting as a chain initiator [37,41]), leading to the formation of pyrolytic carbon deposits appearing as few-layer graphene structures. Thus, bithiophene pyrolysis opens a route for the cheap production of hierarchical composite thin films comprising carbon nanotubes and few-layer graphene, which might be of practical use for hierarchical adsorbents, protective membranes, or electrocatalysis.

## Data Availability

Not applicable.
